# Impact of continuous positive airway pressure treatment on left atrial volume and function in patients with obstructive sleep apnoea assessed by real-time three-dimensional echocardiography

**DOI:** 10.1136/hrt.2009.173625

**Published:** 2009-07-29

**Authors:** W Oliveira, O Campos, F Cintra, L Matos, M L C Vieira, B Rollim, L Fujita, S Tufik, D Poyares

**Affiliations:** 1 Discipline of Sleep Biology and Medicine, Department of Psychobiology, Federal University of Sao Paulo, Sao Paulo, Brazil; 2 Albert Einstein Hospital, Sao Paulo, Brazil; 3 Discipline of Cardiology, Federal University of Sao Paulo, Sao Paulo, Brazil

## Abstract

**Background:**

Obstructive sleep apnoea (OSA) has been reported as a predictor of left ventricle (LV) diastolic dysfunction and left atrium (LA) remodelling. The aim of this study is to evaluate the impact of OSA treatment with a continuous positive airway pressure device (CPAP) on the LA volume and function, as well as on the LV diastolic function.

**Methods:**

In total, 56 OSA patients were studied. All patients underwent real-time three-dimensional (RT3DE) and two-dimensional echocardiogram with tissue Doppler evaluation in order to estimate LA volumes, function and LV diastolic performance. A total of 30 patients with an apnoea-hypopnoea index greater than 20 were randomly selected to receive sham CPAP (n = 15) or effective CPAP (n = 15) for 24 weeks. They underwent echo examination on three different occasions: at baseline, after 12 weeks and 24 weeks of CPAP or sham CPAP.

**Results:**

In the effective CPAP group we observed the following changes from the baseline to the 24-week echo evaluation: (a) a reduction in the E/E′ ratio (10.3 (1.9) to 7.9 (1.3), p = 0.03); (b) an increase in the LA passive emptying fraction (28.8% (11.9%) to 46.8% (9.3%), p = 0.01); and (c) a reduction in the LA active emptying fraction (42.7% (11.5%) to 25.7 (15.7), p<0.01). In the sham group, there were no changes from the baseline to the 24-week echo. We found a positive correlation between 24 week/baseline LA active emptying volume and 24 week/baseline E/E′ ratios (r = 0.40, p<0.05) and a negative correlation between 24 week/baseline LA passive emptying volume and 24 week/baseline E/E′ ratios (r = −0.53, p<0.05). No significant changes were found on LA total emptying fraction.

**Conclusion:**

CPAP improved LV diastolic function and LA passive emptying, but not LA structural variables in OSA patients.

**Trial registration number:**

NCT00768807.

Obstructive sleep apnoea (OSA) is a prevalent condition in the general population, and it is characterised by recurrent upper airway obstruction during sleep, which results in periods of apnoea/hypopnoea, oxyhaemoglobin desaturation, abnormal nocturnal arousals and daytime sleepiness.^1^ It is also described as an important factor of increasing cardiovascular morbidity and mortality. Several studies have linked OSA to increased risk of stroke, hypertension and death.^2^ Notwithstanding, the majority of risk factors for OSA, such as obesity, male gender and ageing, are also related to cardiovascular disorders, which makes it difficult to define OSA as an independent risk factor.^3^

Studies have reported a higher frequency of diastolic dysfunction in OSA patients when compared to controls.^4^ The reduction of left ventricle (LV) compliance might result in left atrium (LA) overstretching and dilation in order to maintain proper LV filling. Diastolic dysfunction and LA enlargement have been associated with several clinical conditions, including hypertension, diabetes, left ventricular systolic dysfunction, obesity and ageing.^5^ ^6^ In addition, according to epidemiological studies, LA enlargement is an independent predictor of major cardiovascular events.^7^

The association between OSA and LA volume augmentation has been previously studied and seems to be related to OSA severity. Diastolic dysfunction is also likely to be attached to LA functional and structural remodelling.^8^ ^9^ However, the impact of the effective OSA treatment with long-term continuous positive airway pressure on LA function and structure has never been analysed using real-time three-dimensional echocardiography (RT3DE).

We hypothesised that the morphological and functional LA changes that occur in patients with OSA could be at least partially reversed after the effective OSA treatment with a chronic continuous positive airway pressure device (CPAP). Therefore, we sought to evaluate the LA volume and function, as well as LV diastolic variables, in moderate to severe OSA patients before and after 24 weeks of effective or sham CPAP.

## Methods

### Population

A total of 56 recently diagnosed patients with mild to severe, untreated OSA (29–70 years, 29 men) were recruited and referred to the sleep laboratory of the Universidade Federal de Sao Paulo (Sao Paulo, Brazil). OSA diagnoses were confirmed by full polysomnography. All patients were first examined by a member of the cardiology staff, and blood pressure was recorded by the auscultatory method. Hypertension was considered according to the seventh report of the Joint National Committee on Prevention, Detection, Evaluation, and Treatment of High Blood Pressure.^10^ Individuals were excluded from both groups on the basis of the following characteristics: body mass index (BMI) >35 kg/m^2^, history of coronary artery disease and atrial fibrillation, cardiomyopathy and poor-quality imaging on two-dimensional echocardiography and/or RT3DE. Other medical conditions, such as severe arrhythmias and pulmonary and valvular heart disease were also excluded.

The research protocol was approved by the institution’s ethics committee, and informed consent was obtained from each patient.

### Polysomnography

Each patient underwent polysomnographic recording, with a minimum duration of 7 hours. Recordings were performed using 16-channel Somnologica Studio Science, (Medcare, Iceland) with a resolution of 200 Hz on two different occasions: (1) baseline polysomnography (before entry) and (2) CPAP titration polysomnography. Two researchers visually scored the recordings according to standard criteria.^11^ ^12^

The variables analysed were total sleep time, sleep efficiency (sleep time/recording time ×100), sleep latency (time from lights-off to sleep onset), REM and non-REM sleep percentage, apnoea-hypopnoea index (apnoea + hypopnoea events per hour), arousals/hour and oxygen saturation. Apnoea was defined as a decrease in airflow of at least 80% for 10 seconds or more, and hypopnoea was defined as a decrease in airflow of at least 50% for 10 seconds or more. An apnoea-hypopnoea index (AHI) >5 was considered diagnostic for OSA.

### Study design and CPAP randomisation

**Figure 1:**
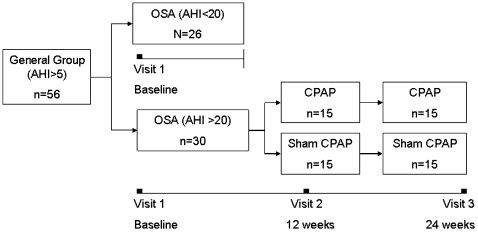
Study design. AHI, apnoea-hypopnoea index; OSA, obstructive sleep apnoea; CPAP, continuous positive airway pressure device.

This is a randomised, double-blinded, placebo-controlled, single-centre study, in which a subsample of 30 patients, selected from the initial general group (n = 56), with AHI >20 were randomly divided to receive placebo (sham CPAP) (n = 15) or effective CPAP for 24 weeks (n = 15). The randomisation of patients with AHI >20 was based on the American Academy of Sleep Medicine recommendation for CPAP treatment.^13^ (fig 1).

Sham CPAP consisted of a modified traditional CPAP, Resmed S8. Modifications were performed on both the equipment and the mask. A swivel including four small holes, comprising a 12-mm^2^ area, was incorporated into the mask close to the tracheal connection. In addition, a specially manufactured silicone cork with a 3.5-mm orifice was inserted in the pressured air output of the CPAP machine. These modifications were aimed at losing pressure, but allowed the sensation of circulating air into the mask. All sham CPAPs were regulated at 10 cm H_2_O and delivered a maximum of 1.5 cm H_2_O of positive pressure, as measured with a vacuum manometer. They were all tested, and end-tidal CO_2_ was assessed in the mask, which was within 35–45 mm Hg during the entire polysomnography titration night for all patients.^12^ Patients were followed every 2 weeks by the medical staff, and compliance and side effects of CPAP were systematically checked. Compliance was considered a minimal usage of 5 hours nightly, as determined by the hour meter. All 15 patients allocated to the sham group received effective CPAP treatment after completion of the protocol.

### Echocardiography

All individuals were submitted to a baseline comprehensive 2D echocardiographic study according to the American Society of Echocardiography guidelines, followed by a RT3DE examination performed by two experienced investigators who were blinded to the polysomnographic results. The randomised patients underwent echo examination on three different occasions: at baseline, after 12 weeks, and after 24 weeks of CPAP treatment or placebo (sham CPAP). A commercially available machine (IE33, Philips Medical Systems, Andover, MA, USA) with digital storage software for offline analysis was used.^14^

RD3DE was performed with an X3 matrix-array transducer (1–3 MHz) for acquisition of “full-volume” real-time pyramidal volumetric data sets along four consecutive cardiac cycles. Individuals were instructed to hold their breath, and images were coupled with electrocardiographic recordings. Apical two-chamber and four-chamber views were extracted from the pyramidal data set during expiration. Both left ventricular and LA cavities were included in the pyramidal scan volume. The RT3DE data sets were digitally stored and analysed using analysis software (QLab-Philips version 4.2; Philips Medical systems). Maximum LA volume, minimum LA volume and LA volume before contraction were obtained.

Anatomical landmarks used to calculate LA volumes were manually identified as follows: lateral, septal, anterior and posterior points of the mitral annulus and the higher point of the LA roof. Points determined to represent the pulmonary vein ostia or LA appendages were excluded from the measurement. The LA internal endocardial border of each frame was defined by automated processing and manually adjusted for pulmonary vein ostia and LA appendage exclusion. From these data, a three-dimensional model of LA volume was generated.

The following LA measures were calculated: (1) total emptying fraction, the difference between maximal volume and the minimum volume divided by the maximum volume; (2) active emptying volume, the difference between the volume before atrial contraction and the minimum volume; (3) active emptying fraction, active emptying volume divided by the volume before atrial contraction; (4) passive emptying volume, the difference between maximal volume and the volume before atrial contraction; and (5) passive emptying fraction, the passive emptying volume divided by the maximum volume.^15^ ^16^ ^17^

The LV ejection fraction was also assessed by RT3DE via evaluation of apical four-chamber and two-chamber views using the pyramidal three-dimensional data set.^18^ Left ventricular diastolic function was assessed with Doppler echocardiography in accordance with the American and European Societies of Echocardiography recommendations.^19^ ^20^ The mitral inflow was recorded with the pulsed-wave Doppler sample volume located between the leaflet tips. The following variables were measured: peak flow velocity in early diastole (E wave), peak velocity at atrial contraction (A wave) and mitral deceleration time. Tissue Doppler imaging of the mitral annulus (maximal diastolic early E′ and late A′ waves derived from averaged velocities of lateral and septal curves). These data were used for calculating the E/E′ ratio. The LV mass index was obtained according to American Society of Echocardiography recommendations, which are based on two-dimensional echocardiographic linear measurements.^21^

### Statistical analysis

Data are presented as mean (SD) or frequencies. One-way ANOVA was used to compare the OSA mild, moderate and severe subgroups with respect to the following parameters: RT3DE, Doppler, polysomnography and demographic variables. Repeated measures ANOVA was used to compare the randomised individuals before and after 12 weeks and 24 weeks of effective CPAP and sham CPAP. The Bonferroni post-hoc test was applied to assess significant differences after CPAP treatment. χ^2^ was chosen to compare the frequencies of categorical data. The level of significance was set at p<0.05. Pearson matrices were used to estimate the correlations between the 24-week/baseline LA active and passive emptying volumes ratios with 24-week/baseline E/E′ ratio.

## Results

### General and polysomnographic results

In total 60 OSA patients were initially invited to participate in this study and 56 were included. One was excluded because of angina symptoms, a second because of abnormal lung function, and the last two because of poor quality imaging on RT3DE. All randomised patients (n = 30) successfully completed the 24 weeks of CPAP or sham CPAP. Full compliance failed in two patients who belonged to the sham group because of non-complicated upper airway infection. They refrained from device usage for a mean of 5 days. Their data were included in the final sample.

Higher frequency of male gender, diabetes and hypertension was found in the severe compared to mild and moderate OSA groups (p<0.05). Mean age, body mass index, neck circumference, heart rate and systolic and diastolic blood pressure did not differ between groups. As expected, the apnoea-hypopnoea index (events/h), microarousal index/h, and O_2_ saturation nadir were significantly different between all groups (p<0.05).

Baseline characteristics and polysomnographic data are displayed in table 1.

**Table 1:** Clinical characteristics and polysomnographic results

	Mild OSA (n = 17)	Moderate OSA (n = 18)	Severe OSA (n = 21)
Age (years)	49.8 (10.8)	53.1 (10.4)	56.8 (9.30)
Male gender (%)	47.1	44.4	61.9*†
Body mass index (kg/m^2^)	28.4 (7.3)	29.2 (5.7)	30.3 (6.2)
Neck circumference (cm)	36.6 (4.1)	37.2 (3.8)	38.8 (3.9)
Hypertension (%)	29.5	33.3	66.7*†
Systolic blood pressure (mm Hg)	140.1 (20.3)	134.5 (18.1)	142.1 (19.5)
Diastolic blood pressure (mm Hg)	88.8 (12.9)	80.7 (11.8)	86.8 (12.7)
Diabetes mellitus (%)	0	0	19.9*†
Heart rate (beats/min)	67.0 (13.4)	60.4 (10.4)	65.3 (9.4)
Sleep efficiency (%)	86.6 (7.5)	79.7 (13.4)	83.5 (12.0)
Total sleep time (min)	362.7 (95.6)	353.8 (71.8)	362.8 (83.2)
Stage 1 (%)	7.8 (10.6)	7.3 (7.3)	6.7 (11.8)
Stage 2 (%)	55.6 (13.6)	46.0 (21.5)	57.7 (18.6)
Slow wave sleep (%)	18.3 (10.1)	27.9 (14.0)	24.8 (16.3)
Rapid eye movement sleep (%)	19.0 (7.6)	20.9 (13.7)	15.4 (10.5)
Apnoea-hypopnoea index (events/h)	9.9 (2.6)	21.1 (3.7)*	54.8 (22.5)*†
Microarousal index/h	12.9 (6.50)	16.6 (6.6)*	25.2 (13.7)*†
O_2_ saturation nadir (%)	87 (3.8)	83.1 (10)	76.1 (0.2)*

*Different from mild, p<0.05; †different from moderate, p<0.05.

### Echocardiographic baseline results

In the initial sample of 56 OSA patients, the severe OSA group showed higher values of E mitral deceleration time and E/E′ ratio compared to the mild OSA group and higher average A′ velocity, 3D maximum, minimum and pre-contraction LA volumes compared to the moderate and mild groups. (p<0.05, all). Also, the severe group presented lower E′ and E′/A′ ratios than the mild and moderate groups.

### CPAP versus sham results

Out of 30 OSA patients who underwent polysomnography for CPAP titration, only 15 who were randomised to the effective CPAP group had successful positive pressure titration, as observed by correction of AHI, oxygen saturation and snoring.

A minimal of 5 hours/night of CPAP usage was ensured in both groups, according to hour-meter analysis, with the exception of two patients in the sham group.

We did not observe any significant differences between the sham CPAP and effective CPAP groups where the baseline demographic and echocardiographic variables were concerned as displayed in table 3.

**Table 2:** Echocardiogram baseline results

	Mild OSA (n = 17)	Moderate OSA (n = 18)	Severe OSA (n = 21)
Left ventricular mass index (g/m^2^)	83.4 (20.0)	83.6 (12.2)	94.0 (27.0)
E velocity (cm/s)	73.8 (17.8)	74.4 (18.9)	68.1 (14.6)
A velocity (cm/s)	64.6 (14.1)	71.2 (19.3)	74.3 (19.5)
E/A ratio	1.2 (0.4)	1.1 (0.3)	1.0 (0.3)
E mitral deceleration time (ms)	189.2 (34.5)	231.8 (45.2)	247.6 (48.2)*
E/E′ ratio	9.7 (1.9)	10.4 (3.1)	11.6 (3.6)*
Averaged E′ velocity (cm/s)	7.7 (1.5)	7.3 (2.0)	6.2 (1.8)*†
Averaged A′ velocity (cm/s)	6.1 (1.4)	6.8 (1.7)	8.2 (1.9)*†
E′/A′ ratio	1.3 (0.4)	1.1 (0.3)	0.8 (0.3)*†
S velocity (cm/s)	7.4 (2.1)	8.1 (2.5)	7.6 (1.7)
3D LA maximum volume (ml)	37.1 (14.7)	40.8 (8.9)	57.6 (31.0)*†
3D LA minimum volume (ml)	13.9 (5.5)	16.6 (4.0)	24.5 (20.9)*†
3D volume before LA contraction (ml)	24.3 (10.2)	24.7 (5.4)	44.2 (28.4)*†

*Different from mild, p<0.05; †different from moderate, p<0.05.

**Table 3:** Effective continuous positive airway pressure (CPAP) treatment and sham groups baseline characteristics

	Effective CPAP (n = 15)	Sham (n = 15)	p Value
Age (years)	56.0 (10.1)	53.1 (10.4)	0.9
Male gender (%)	60.0	46.0	0.4
Body mass index (kg/m^2^)	29.6 (6.2)	30.8 (6.2)	0.6
Neck circumference (cm)	37.9 (4.5)	38.2 (3.5)	0.8
Hypertension (%, n)	53.0 (8)	53.0 (8)	1
Systolic blood pressure (mm Hg)	142.3 (19.8)	138.1 (10.5)	0.5
Diastolic blood pressure (mm Hg)	87.2 (12.1)	81.8 (8.2)	0.2
Diabetes mellitus (%, n)	13.0 (2)	13.0 (2)	1
Heart rate (beats/min)	67.8 (13.4)	68.9 (12.5)	0.8
Sleep efficiency (%)	83.8 (7.5)	86.2 (12.1)	0.5
Total sleep time (min)	358.1 (104.5)	385.2 (49.6)	0.4
Apnoea-hypopnoea index (events/h)	39.2 (21.3)	43.9 (20.1)	0.5
Microarousal index/h	18.9 (9.7)	20.4 (9.6)	0.7
O_2_ saturation nadir (%)	77.4 (18.3)	79.2 (10.4)	0.8
Left ventricular mass index (g/m^2^)	82.5 (17.6)	85.6 (14.7)	0.6
E velocity (cm/s)	70.7 (13.2)	60.6 (25.2)	0.6
A velocity (cm/s)	69.9 (15.8)	75.1 (21.1)	0.5
E/A ratio	1.0 (0.2)	1.0 (0.4)	0.6
E mitral deceleration time (ms)	227.0 (38.8)	244.2 (21.3)	0.1
E/E′ ratio	10.3 (1.9)	10.5 (2.6)	0.8
Averaged E′ velocity (cm/s)	7.0 (1.5)	6.6 (1.9)	0.6
Averaged A′ velocity (cm/s)	7.8 (2.3)	7.1 (1.6)	0.3
E′/A′ ratio	1.0 (0.4)	1.0 (0.3)	0.9
S velocity (cm/s)	8.2 (1.9)	7.8 (2.3)	0.5
3D LA maximum volume (ml)	45.2 (11.9)	46.2 (17.3)	0.9
3D LA minimum volume (ml)	18.0 (5.1)	18.6 (6.4)	0.8
3D volume before LA contraction (ml)	32.1 (9.5)	30.8 (13.6)	0.8

**Figure 2:**
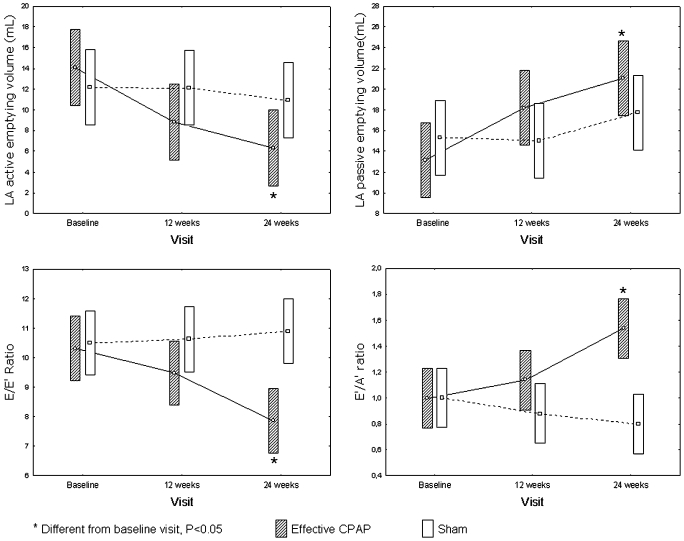
Left atrial (LA) function and left ventricular diastolic performance throughout 24 weeks of sham or effective continuous positive airway pressure (CPAP) treatment.

However, when baseline echo variables were compared to the 12-week and 24-week evaluation of both treatments, we observed only in the CPAP effective treatment group: (a) an increase in the E′/A′ ratio (1.0 (0.4) to 1.5 (0.7), p = 0.02); (b) a reduction in the E/E′ ratio (10.3 (1.9) to 7.9 (1.3), p = 0.03); (c) an increase in the E′ averaged mean velocity (7.0 cm/s (1.5) to 9.5 cm/s (1.9), p<0.01); (d) an increase in the LA passive emptying volume (13.2 ml (6.1) to 21.0 ml (7.4), p = 0.04); (e) an increase in the LA passive emptying fraction (28.8% (11.9%) to 46.8% (9.3%), p = 0.01); (f) a reduction in the LA active emptying volume (14.0 ml (6.5) to 6.3 ml (4.3), p = 0.05); and (g) a reduction in the LA emptying ejection fraction (42.7% (11.5%) to 25.7% (15.7%), p<0.01) (fig 2). There were no significant differences after 12 weeks of CPAP or during the entire sham usage in respect to these variables.

Additionally, there was a trend towards a reduction of the E/A ratio from baseline to 24-week echo evaluation in the effective CPAP group (1.0 (0.2) to 1.4 (0.1), p = 0.06).

We did not find a significant difference in the LV mass index throughout the 24-week CPAP therapy between the baseline and 24-week echo evaluation (82.5 g/m^2^ (17.6 vs 86.7 g/m^2^ (18.5, NS). Other echocardiographic variables and clinical data such as LV ejection fraction, LA maximal, minimum and pre-contraction fundamental volumes, body mass index, blood pressure and heart rate did not significantly change in both effective CPAP and sham groups, at any time.

**Figure 3:**
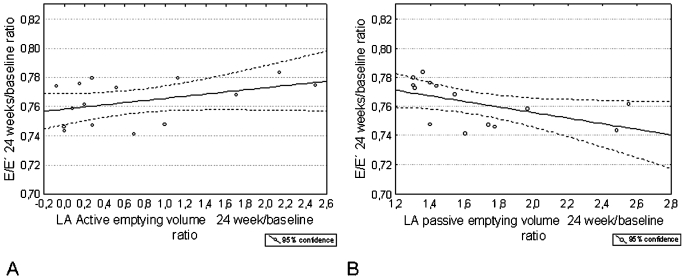
(A) Correlation between 24-week/baseline left atrium (LA) active emptying volume and 24-week/baseline E/E′ ratios. (B) Correlation between 24-week/baseline left atrium (LA) passive emptying volume and 24-week/baseline E/E′ ratios.

Pearson matrices showed a positive correlation between 24-week/baseline LA active emptying volume and 24-week/baseline E/E′ratios (r = 0.40, p<0.05) and a negative correlation between 24-week/baseline LA passive emptying volume and 24-week/baseline E/E′ ratios (r = −0.53, p<0.05) (fig 3).

## Discussion

The main finding of this study is the significant enhancement of LA passive function, as noticed by the increment of LA passive emptying and reduction of LA active function after 24 weeks of CPAP treatment. This finding seems to be directly associated with the improvement of LV diastolic function after the effective treatment of OSA. To the best of our knowledge, this is the first study which analyses the effects of CPAP on the LA dynamics and LV diastolic function using RT3DE and tissue Doppler technologies. In addition, the randomised placebo-controlled intervention group with similar demographic variables, supports the idea that OSA contributes significantly to left atrial dysfunction

The association of LV diastolic impairment and OSA has been studied, but has not yet been fully explained. The possible explanations are constant catecholaminergic hyperactivity, a rise in the negative intrathoracic pressure and many conditions highly prevailing in OSA, such as hypertension, obesity and ageing, which act in synergy to result in LV diastolic burden and myocardial relaxation impairment.^22^ ^23^ We observed impaired diastolic function in the severe OSA group compared to mild and moderate OSA groups, when analysing the E/E′ and E′/A′ ratios. The poor diastolic function in OSA patients is in agreement with the study of Arias *et al*, which used pulse Doppler.^24^

Alterations in LA volumes and function could be linked to diastolic impairment in patients with OSA. A reduced passive LA emptying results in a less significant motion of the mitral valve annulus, which can be shown by the reduced velocity of the E′ wave. The impairment of LA passive emptying also contributes to a larger residual LA volume before its active contraction. According to the landmark Frank-Starling study, there is an augmentation of LA contraction force due to LA pre-systolic volume and fibre length increase. The atrial contraction becomes of crucial importance during LV filling, as suggested by the higher values of 3D LA active emptying volume and 3D LA active emptying fraction in the severe OSA group. These findings are in accordance with the increased velocity of A′ in that group. In our opinion, the constant atrial overload ultimately plays an important part in the augmentation of maximum LA volume in OSA patients.^25^ ^26^

The partial improvement of diastolic function after long-term treatment with CPAP supports a physiopathological association between diastolic function and OSA. Some non-controlled studies using different criteria for selection have found changes in diastolic variables after CPAP.^27^ ^28^ The CPAP would lead to a reduction in the exaggerated negative intrathoracic pressure, which, in turn, would lead to a reduction in apnoeic-hypopnoeic events and, therefore, in the ventricular afterload.^29^

The reduction in the active LA emptying and the increase in the LA passive emptying (fig 2) reflect the observed changes in LV relaxation dynamics. This finding is reinforced by a significant linear positive correlation between 24-week/baseline LA active emptying volume and 24-week/baseline E/E′ ratios and by the negative correlation between 24-week/baseline LA passive emptying volume and 24-week/baseline E/E′ ratios (fig 3).

Although we did not observe significant differences in absolute LA volumes between baseline and 24-week echo examination in the CPAP group, LA passive emptying volume and passive emptying fraction increased. On the other hand, LA active emptying volume and LA active emptying fraction significantly increased after CPAP. This finding reveals an atrial functional remodelling which could anticipate a LA structural modification.

In addition, another potential novelty of this study is the concept that the relation between LA active and passive emptying, as assessed by RT3DE, could be a sensitive indicator for LA work and reflects the severity of LV diastolic function.

Precision in the estimate of LA size is necessary for clinical practice owing to the association between increased LA volume and major adverse cardiovascular outcomes. LA analysis using RT3DE has been validated as a prognostic mark of major cardiac events, and shows potential in terms of accuracy, practicability and reproducibility.^15^ ^16^ This technology has already been used to study LA structure in patients with OSA, and OSA severity was shown to be an independent predictor of LA maximal enlargement.^8^

In summary, our results do not simply highlight the impact of OSA treatment on diastolic function, as seen in previous studies which used pulse-Doppler technology, but also reveal the effect of CPAP on the LA functional performance in this population. The repercussion of a longer CPAP therapy on LA structure and absolute volumes, as well as its potential impact on the development or recurrence of atrial rhythm disturbances, need to be investigated further.

### Study limitations

The main potential limitation was the presence of hypertension in this population and its influence on diastolic function. We may not have found a significant decrease in blood pressure, probably because we recorded it on a by-office basis instead of by 24-hour ambulatory blood pressure monitoring. In fact, our purpose was to study the ultimate effect of CPAP on LA function in a commonplace OSA population, with its typical comorbidities. Moreover, a previous study showed that OSA severity is an independent predictor of LA functional and structural remodelling when corrected for hypertension, BMI, gender and diabetes.^8^

## Conclusions

We demonstrated, using RT3DE, that the functional LA burden observed in OSA could be, at least partially, reversed after effective long-term CPAP therapy. This finding seems to be strongly associated with the improvement of diastolic function in this population after correction for apnoeic-hypopnoeic events.
